# Opportunities of measuring hierarchical models of psychopathology

**DOI:** 10.1002/jcv2.12187

**Published:** 2023-07-22

**Authors:** Erik Pettersson

**Affiliations:** ^1^ Department of Medical Epidemiology and Biostatistics Karolinska Institutet Stockholm Sweden

**Keywords:** comorbidity, general psychopathology, hierarchical factor models, P factor

## Abstract

All psychiatric phenomena are positively associated, and several different models can account for this observation. These include the correlated factors, network, general psychopathology as outcome, and hierarchical models. Advantages of hierarchical models, which consist of one general and several (general factor‐residualized) specific factors, is that the general factor provides an opportunity to reliably measure global distress and impairment, while the specific factors might improve the ability to discriminate between individuals with different kinds of problems. Nevertheless, other models also have their respective advantages, and it remains challenging to empirically determine which model best accounts for the positive manifold in psychiatry. Instead, I present two non‐empirical arguments in favor of hierarchical models. First, by measuring the general factor in isolation, the specific factors tend to include both favorable and unfavorable correlates, which might reduce stigma compared to psychiatric diagnoses that by and large are associated with only unfavorable outcomes. Second, the general psychopathology factor displays an unusual psychometric property in that it includes symptoms of opposite meaning if they have similar valence (e.g., self‐reported symptoms such as *gullible* and *paranoid*, *lazy* and *workaholic*, and *terrified* and *apathetic* load in the same direction), which one might want to measure in isolation from variance capturing the content of symptoms. I conclude by speculating that tests designed based on hierarchical models might help clinical assessment.

## Graphical abstract

Several models can account for the observation that all psychiatric phenomena are positively associated. I present two non‐empirical arguments in favor of hierarchical models, which I suggest might help improve clinical assessment.

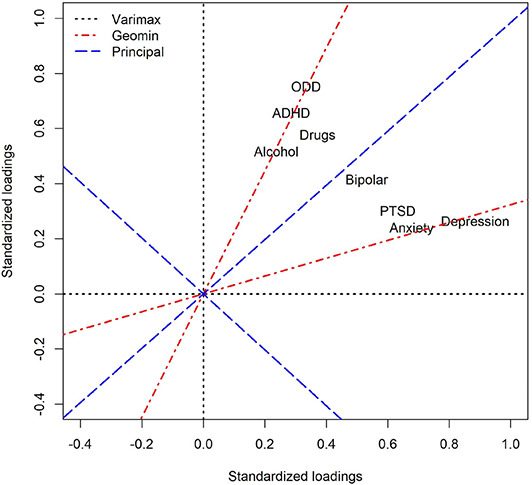

**Key points**

- Network, correlated factors, general factor as outcome, and hierarchical models can all account for the observation that all psychiatric phenomena are positively associated
- Advantages of hierarchical models include that they offer the opportunity to reliably measure broad distress and impairment while potentially improving discriminant validity
- Two non‐empirical arguments in favor of measuring hierarchical models is that the (general factor‐residualized) specific factors might reduce stigma surrounding mental health problems, and that the general factor displays an unusual psychometric property such that it might be worthwhile to measure in isolation

## INTRODUCTION

Regardless of whether based on surveys in community samples or psychiatric diagnoses assigned following contact with the mental health system, individuals who suffer one symptom, syndrome, or psychiatric disorder are more likely to suffer all other related conditions (Barr et al., 2022; Kessler et al., 2005; Lahey et al., 2014; Plana‐Ripoll et al., 2019). The magnitude of the overlap among psychiatric phenomena is similar to the magnitude of the overlap among intelligence subtests (Pettersson et al., 2020). Furthermore, when psychological problems are combined simultaneously in multiple regression models that predict future functional impairment, the unique associations often tend to the null, highlighting that a substantial portion of the predictive power might be attributable to shared rather than unique symptom variance (Copeland et al., 2015; Kessler et al., 2012; Pettersson et al., 2018). However, regression models do not provide an estimate of the effect attributed to shared variance.

Psychologists have recently suggested that the overlap among psychological problems might be accounted for by a hierarchical model consisting of a general psychopathology factor that account for covariation among all symptoms, and several (general factor‐residualized) specific factors that account for covariation unique to subsets of symptoms (Caspi & Moffitt, 2018; Lahey et al., 2017). In contrast to multiple regression models, several parameterizations of hierarchical factor models allow for estimating associations between shared variance and future clinically relevant outcomes (Lahey et al., 2021; Pettersson et al., 2021). Research shows that general psychopathology uniquely predicts adverse outcomes above and beyond specific syndromes. These associations persist even when the raters differ, minimizing potential attributions to shared rater biases (Laceulle et al., 2020; O'Reilly et al., 2020; Pettersson et al., 2018), and the magnitudes rival those between adolescent general intelligence and adult educational attainment (Pettersson et al., 2020).

### Models consistent with the positive manifold in psychiatry

In addition to hierarchical models, several other models are also consistent with the observation that all psychiatric phenomena are positively associated. Below I briefly review three such models.

#### Correlated factors model

An advantage of the correlated factors model is that it tends to produce factors that are intuitive to interpret (e.g., the Big Five model of personality, or the Internalizing and Externalizing psychopathology dimensions). A disadvantage of this model, as I will review below, is that it tends to poorly discriminate between individuals with different mental health problems. In addition, from a causal inference perspective, correlations among latent variables beg the question of their origin. One possibility of course is that such dimensions (e.g., the internalizing and externalizing spectra) causally increase risk of the other, but this assumption is not always made explicit.

#### Network models

Inspired by zoology where different organisms interact to create complex ecological systems, network models assume that there are no common causes (i.e., no latent variables influencing several indicators); instead, all symptoms and disorders are postulated to cause each other. An advantage of this model is that it allows for estimating causal effects among symptoms. For instance, it seems reasonable that sleep problems might have deleterious downstream effects on inattention symptoms, above and beyond that which they might share due a latent depression factor.

Nevertheless, network models also make several assumptions that may or may not be true. First, they assume that there is no measurement error. This would imply that one can measure depression and height with equal precision, which might be unrealistic. Second, they assume that that there is no unmeasured confounding. Given that virtually all psychiatric phenomena are genetically correlated (Grotzinger et al., 2022), assuming zero genetic confounding might be unrealistic. Third, as will be reviewed below, perceived item valence appears to be an important factor in self‐report data (Edwards, 1969). If a network model were applied to a questionnaire where items were balanced for perceived valence, it might end up with putative causal paths between items such as *gullible* and *paranoid*, *lazy* and *workaholic*, and *terrified* and *apathetic*, etc., which might be unrealistic.

#### General psychopathology as an outcome of specific factors

General psychopathology as an outcome of specific factors is also consistent with the observation that all psychiatric phenomena are positively associated. That is, internalizing and externalizing spectra might cause general psychopathology, rather than the other way around. While it is challenging to estimate directionality, one can tentatively begin to try to infer it by comparing how well observed data match different data generating mechanisms. Below I apply three such approaches.

First, if general psychopathology were an outcome, one might expect that the variance accounted for by the general factor (i.e., its magnitude) should increase over time. However, in three cross‐sectional samples of children, adolescents, and adults, the magnitude of general psychopathology was similar (Pettersson et al., 2020). Likewise, in a longitudinal sample of children living in Switzerland who were rated by teachers on 39 symptoms at eight occasions between ages 7 and 15, a total sum score was roughly equally associated with latent general psychopathology across the eight measurement occasions (Murray et al., 2016). Similarly, in a longitudinal sample of U.S. children rated by their mothers on various symptoms, general psychopathology accounted for a similar degree of variance at each wave (McElroy et al., 2018). More recently, in a longitudinal sample of U.S. girls who self‐reported on symptoms annually between ages 14–21, a general psychopathology factor appeared similar in magnitude when estimated via exploratory factor analysis (however, it increased in magnitude when estimated via confirmatory factor analysis; Choate et al., 2022). Thus, to date, the magnitude of general psychopathology appears relatively stable from childhood to adulthood, potentially at odds with conceptualizing it as an outcome.

**FIGURE 1:**
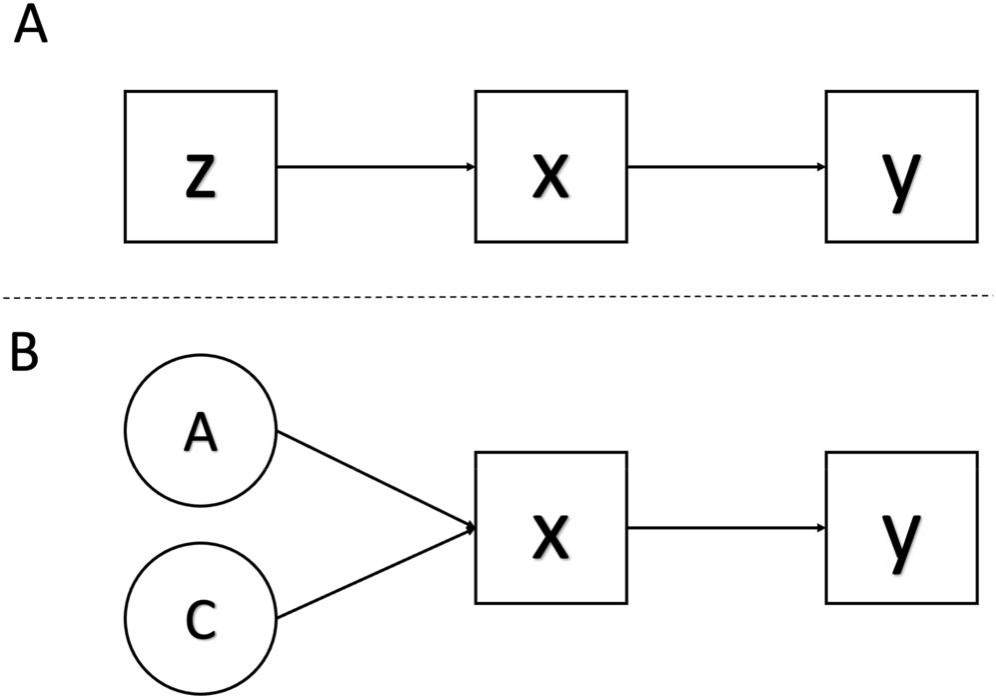
(A) Causal diagram of conditioning on a descendant (Z) of an exposure. (B) Causal diagram of conditioning on two descendants (A and C) of an exposure.

Second, from a causal inference perspective, if lower‐order psychopathology dimensions were to cause general psychopathology, then those associations should not change if we adjust for variables that cause the lower‐order factors. That is, if one adjusts for variable z in Figure 1A by regressing y on both x and z in a multiple regression, then the association between x and y will not change (Appendix S1 displays the corresponding covariance algebra; Pearl, 2013). If we assume that the internalizing and externalizing spectra are partly accounted for by genetics (i.e., they are heritable) and the shared environment, then adjusting for these via a co‐twin control design should not change the association between the internalizing and externalizing spectra and later general psychopathology (Figure 1B). To examine this, I analyzed data on 1480 twin pairs from the Child and Adolescent Twin Study of Adolescent Development (TCHAD), where parents rated their children on the Child Behavior Check‐List (CBCL) at ages 8, 13, 16, and 18, and the children self‐reported on the same measure at ages 13, 16, and 18 (Lichtenstein et al., 2007). The exposures were (parent‐rated) internalizing and externalizing sum scores, and the outcome was (self‐rated) total score (i.e., sum of internalizing and externalizing items) at a later wave. I conducted these multiple regressions in the full sample, as well as within dizygotic (i.e., adjusting for 50% of heritability and 100% of the shared environment) and monozygotic twin pairs (i.e., adjusting for 100% of heritability and 100% of the shared environment). As displayed in Table 1, the associations between internalizing and externalizing problems and later total scores changed within twin pairs, which one would not expect if general psychopathology were an outcome of internalizing and externalizing problems.

**TABLE 1:** Regressing self‐rated total problems on prior parent‐rated internalizing and externalizing dimensions.

	Model		
Regression	Entire sample	Within DZ twin pairs	Within MZ twin pairs
Parent internalizing scale wave 1 ‐> self total score wave 2	0.10 (0.03)	0.18 (0.06)	0.08 (0.10)
Parent externalizing scale wave 1 ‐> self total score wave 2	0.19 (0.03)	0.07 (0.04)	0.04 (0.06)
Parent internalizing scale wave 2 ‐> self total score wave 3	0.22 (0.03)	0.37 (0.08)	0.00 (0.10)
Parent externalizing scale wave 2 ‐> self total score wave 3	0.14 (0.03)	0.04 (0.05)	0.18 (0.07)
Parent internalizing scale wave 3 ‐> self total score wave 4	0.24 (0.04)	0.20 (0.09)	0.04 (0.15)
Parent externalizing scale wave 3 ‐> self total score wave 4	0.09 (0.03)	0.05 (0.07)	0.12 (0.11)

*Note*: Regressions included both the internalizing and externalizing scales as simultaneous predictors. All beta estimates are standardized. Parentheses contain standard errors. Wave 1 = age 8; wave 2 = age 13; wave 3 = age 16; wave 4 = 18. Wave 1 correlation between internalizing and externalizing *r* = 0.50; wave 2 correlation between internalizing and externalizing *r* = 0.56; wave 3 correlation between internalizing and externalizing *r* = 0.45.

**FIGURE 2:**
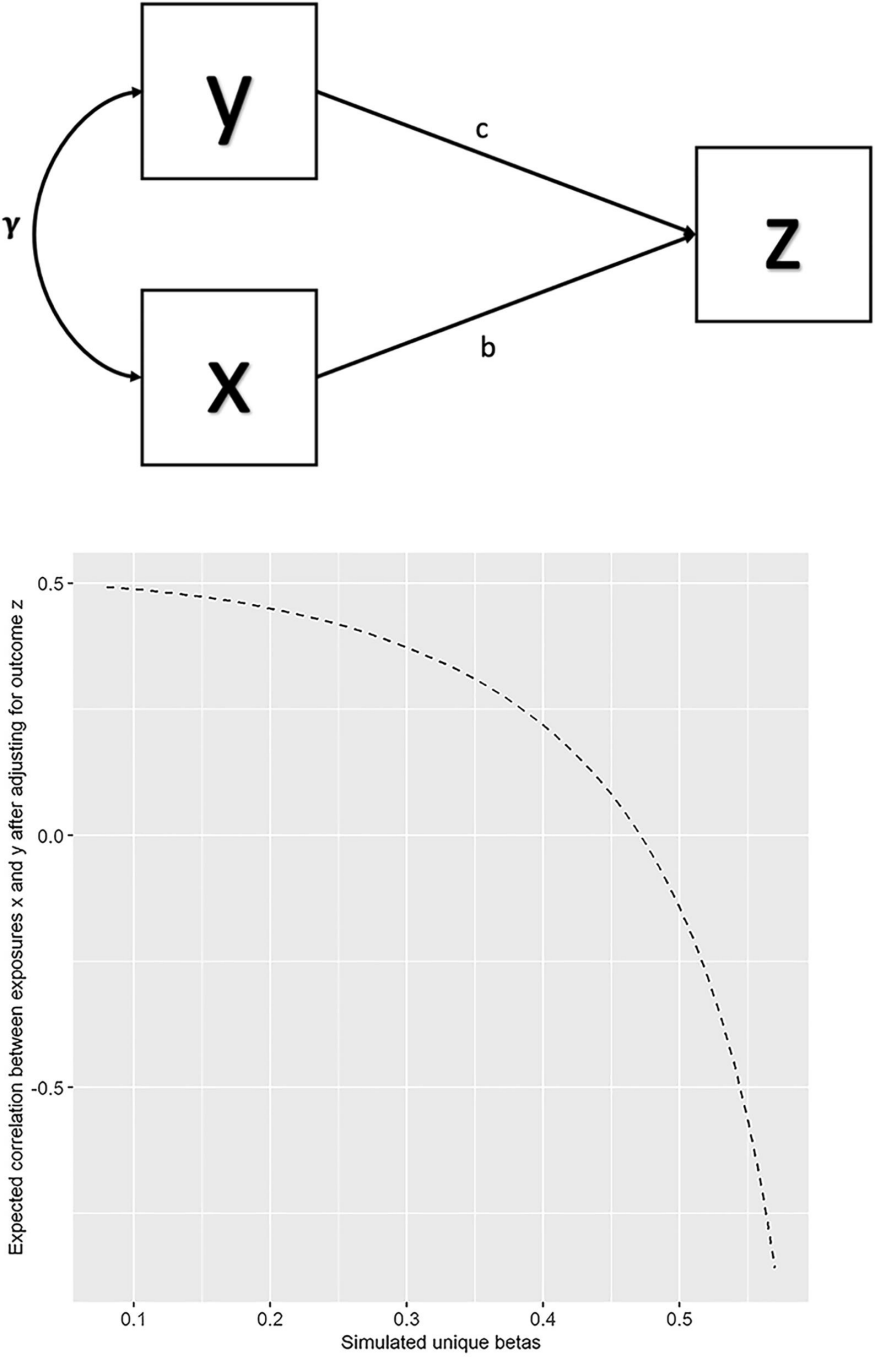
(A) Casual diagram of a collider (z). (B) Changes to the correlation between variables x and y after adjusting for variable z.

Third, assuming that general psychopathology is an outcome implies that it is a collider, such that it is a closed pathway that does not contribute to the covariance between its causes. For example, in the casual graph in Figure 2A if, the variables x and y were to cause the outcome z, then z does not contribute to the covariance between x and y. However, if one were to stratify on or adjust for z, then the pathway via z opens and contributes to the covariance between x and y (Pearl, 2013). Assuming Figure 2A captures the causal structure, Figure 2B shows how an unadjusted correlation between x and y equaling *r* = 0.50 would change when adjusting for outcome z over an iterated range of unique beta weights (Appendix S2 displays the corresponding algebra). For example, the unique betas in the above TCHAD analyses ranged between 0.09 and 0.24, values which would only minimally change the x‐y correlation when adjusting for z according to Figure 2B. One way to adjust for general psychopathology might be to exclude individuals without psychiatric diagnoses (i.e., adjustment via stratification). Watts and colleagues observed that the correlation between the internalizing and externalizing spectra equaled around *r* = 0.50 in three epidemiological samples, but that these correlations dropped to slightly below zero after excluding individuals without psychiatric disorders (Watts et al.). If the internalizing and externalizing spectra were to cause general psychopathology akin to Figure 2A, then the corresponding unique betas would have to be around 0.50 (Figure 2B). As the unique betas in the TCHAD analyses were substantially lower, this large degree of attenuation following stratification might not be consistent with conceptualizing general psychopathology as an outcome. Nevertheless, because there is a scarcity of focus on this topic, more research seems warranted.

### Opportunities of measuring hierarchical models

Whereas all models have their respective advantages, I propose that hierarchical models offer two unique opportunities. First, the general factor in hierarchical models offers the opportunity to reliably measure patients' overall distress and impairment. Although the Global Assessment of Functioning (GAF) index served this purpose in the Diagnostic Statistical Manual (DSM)‐IV, it was dropped in the fifth version because of low reliability (American Psychiatric Association, 2013). In contrast to the GAF index, general psychopathology can be measured with high reliability (Forbes et al., 2021; Moore et al., 2020), so it could assist clinicians when formulating management plans and predicting prognosis.

Second, the specific factors that measure problems independently of general psychopathology in hierarchical models might better discriminate between individuals with different kinds of problems. The challenge of discriminant validity has lingered for decades in psychiatry but has never been adequately resolved. For instance, already back in 1965, Block conducted a detailed psychometric evaluation of the Minnesota Multiphasic Personality Inventory (MMPI) mental health questionnaire, and observed that 355 of the 566 items significantly differed between individuals who scored high versus low on the first principal component (which can be considered synonymous with a general factor; Block, 1965). He concluded that the “MMPI‐item pool heavily weights a first factor relating essentially to adjustment.” (pp. 120), and recommended that “it would be desirable to evolve an item pool which diminishes the pervasiveness of the first MMPI factor.” (pp. 120).

Some forty years later, MMPI test developers heeded this advice by creating the Restructured clinical scales (Tellegen et al., 2003). They noted that the MMPI “general factor appears to inflate correlations between attributes that are considered relatively independent”, and that it would be beneficial to “remove from each Clinical Scale items primarily marking Demoralization” (their label for general psychopathology) to improve the ability to discriminate between patients' problems (pp. 12). However, they were not entirely successful because the mean correlation between Demoralization and the other Restructured scales in the standardization sample equaled *r* = 0.41. Furthermore, the mean correlation among the Restructured clinical scales (excluding Demoralization) equaled *r* = 0.40 whereas the mean correlation among the original clinical scales equaled *r* = 0.47 (Tellegen et al., 2003). In other words, individuals scoring high on one MMPI scale likely also scored high on the other scales, Restructured or not.

A similar issue appears to have surfaced with the introduction of the alternate model for personality pathology in the DSM‐5. This model includes both core impairments in self and interpersonal functioning that are presumably shared by all personality disorders (akin to the MMPI Demoralization scale), as well as maladaptive personality traits (akin to the remaining MMPI Restructured clinical scales). The maladaptive traits were partly developed to improve upon the poor discriminant validity of the DSM‐IV personality disorders (Grant et al., 2005; Skodol et al., 2011; Zimmerman et al., 2005), and can be measured with the Personality Inventory for DSM‐5 (PID‐5; Markon et al., 2013). Mirroring the overlap between the MMPI Demoralization and Restructured clinical scales, in a sample of 365 undergraduate students, the mean correlation between self‐reported personality core impairment and the PID‐5 traits equaled *r* = 0.43 (Sleep et al., 2020). Furthermore, in an online sample of 302 individuals who claimed to have been treated for mental health problems, the mean correlation among the PID‐5 scales equaled *r* = 0.55, whereas the corresponding mean correlations among the ten DSM‐IV personality disorders equaled *r* = 0.42 and *r* = 0.58 based on two different self‐report questionnaires, respectively (McCabe & Widiger, 2020). The authors concluded that “perhaps most surprising and/or disappointing, the current study did not find any improvement to discriminant validity when the analyses were confined to the domains of the dimensional trait model.”(pp. 1167) On a similar note, when individuals with personality disorders self‐report on a Big Five instrument measuring normal personality traits, discriminant validity appears low because all such individuals tend to score high on neuroticism and low on agreeableness and conscientiousness (Morey et al., 2000, 2002).

I propose that one reason for why the MMPI Restructured scales, the PID‐5 inventory, and the Big Five struggle at discriminating between patients with different kinds of psychological problems is because of rotation to simple structure. Because this is a somewhat technical issue, I proceed with an example.

### Rotation to simple structure

After developing multiple factor analysis, Thurstone faced an interpretive problem: the factors could be placed anywhere in the multivariate space yet still reproduce the data equally well. As an analogy, although latitude and longitude assist with navigation, any rotation of these dimensions could be used for the same purpose with equal precision. Therefore, Thurstone invented the concept of simple structure, which involves rotating the factors so that they align with clusters of indicators in the multivariate space (Thurstone, 1947). Whereas this was initially done manually in an arduous procedure, nowadays there are many algorithms that automatically satisfy Thurstone's criteria with either uncorrelated (or orthogonal, such as Varimax) or correlated (or oblique, such as Geomin) factors.

**FIGURE 3:**
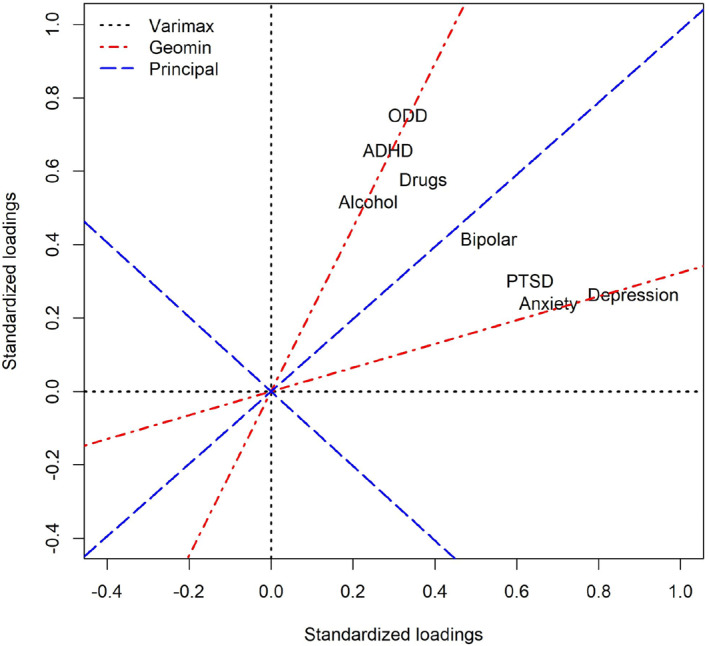
Three rotations of an exploratory factor analysis of eight psychiatric disorders.

To illustrate, I re‐analyzed 414,595 Swedish males born 1980–1992 (Pettersson et al., 2020). Briefly, we examined whether they had been diagnosed with psychiatric diagnoses (depression, anxiety, post‐traumatic stress disorder [PTSD], bipolar disorder, alcohol misuse, drug abuse, attention‐deficit hyperactivity disorder [ADHD], and oppositional defiant disorder [ODD]) prior to the mandatory conscription evaluation (mean age at conscription = 18.3 years old). The Eigenvalues for the eight diagnoses were 3.95, 1.06, 0.90, 0.64, 0.49, 0.43, 0.32, and 0.22, so we extracted two exploratory factors that fit the data well (Root mean square error of approximation = 0.005, 90% confidence interval = 0.004, 0.005; Comparative fit index = 0.983; Tucker‐Lewis index = 0.963; *χ* ^2^ = 127.771, degrees of freedom = 13, *p* < 0.001). Figure 3 displays that two simple structure rotations identified an internalizing and an externalizing factor (Table 2 displays the corresponding factor loadings).

**TABLE 2:** Three rotations of an EFA of eight psychiatric disorders and the corresponding correlations with future prescription of antidepressant medication.

	Rotation					
	Varimax		Geomin		Principal component	
Diagnosis	Internalizing	Externalizing	Internalizing	Externalizing	General	Internalizing versus externalizing
Depression	**0.88**	0.26	**0.94**	−0.03	**0.81**	**0.44**
Anxiety	**0.68**	0.24	**0.70**	0.02	**0.65**	**0.31**
PTSD	**0.63**	**0.30**	**0.61**	0.12	**0.66**	0.23
Bipolar	**0.53**	**0.41**	**0.43**	**0.31**	**0.67**	0.08
Alcohol	0.24	**0.52**	0.01	**0.57**	**0.53**	−0.20
Drugs	**0.37**	**0.57**	0.14	**0.58**	**0.67**	−0.15
ADHD	0.29	**0.66**	−0.01	**0.58**	**0.67**	−0.27
ODD	**0.33**	**0.75**	0.00	**0.83**	**0.77**	**−0.30**
Correlation with later antidepressants	0.43 (0.01)	0.16 (0.02)	0.44 (0.01)	0.31 (0.01)	0.41 (0.01)	0.18 (0.02)

*Note*: Loadings equal to or greater than |0.30| are bolded for visual clarity. Standard errors in parentheses.

Abbreviations: ADHD, Attention‐deficit/hyperactivity disorder; EFA, Exploratory factor analysis; ODD, Oppositional‐defiant disorder; PTSD = Post‐traumatic stress disorder.

I then examined how the internalizing and externalizing factors were associated with anti‐depressant prescription up to 11 years later. As can be seen in Table 2, both factors rotated toward simple structure were positively correlated with later anti‐depressant prescription. This occurs because the outcome inhabits the same area of the multivariate space such that any rotation of the axes that fan out within this quadrant will be positively associated with the outcome, highlighting the challenge the discriminant validity in psychiatry.

One way to improve discriminant validity is to use oblique rotations and multiple regression models, which identify unique associations between predictors and outcomes (cf. Morey et al., 2022). Another way is to rotate the solution toward a hierarchical model by isolating a general factor and setting the specific factors unrelated to general psychopathology by, for example, rotating the dimensions to the first and second principal component (PC) displayed in blue in Figure 3. As can be seen in Table 2, the first PC, on which all disorders loaded in the same direction, was positively associated with later antidepressant prescription. The second PC, which included positive loadings on internalizing diagnoses and negative loadings on externalizing diagnoses, was positively associated with anti‐depressant prescription. That is, among individuals with identical scores on the first PC (i.e., general psychopathology), those prone toward internalizing problems were more likely to be prescribed later antidepressants, whereas those prone toward externalizing problems were less likely to be prescribed antidepressants. Thus, in contrast to the simple structure rotations, the second PC displayed greater discriminant validity, which occurred because the second PC runs perpendicular to the upper left quadrant.

### Two non‐empirical arguments for rotating multivariate solutions toward a hierarchical model

Although all rotations are mathematically equivalent in that they reproduce the observed covariance matrix equally well, one might still argue that Thurstone's simple structure concept represents the best guidelines for factor rotation. However, self‐reported personality pathology data display a high degree of complexity, such that it offers relatively weak guidelines for the placement of the dimensions (Pettersson & Turkheimer, 2014). Similarly, when I applied an algorithm designed to evaluate degree of simple versus complex structure (O'Connor, 2022) to psychiatric conditions in child, adolescent, and adult samples, distinct clusters did not appear to form in the multivariate space (See Appendix S3 and Tables S1–S3). Instead of relying on simple structure rotations, I present two non‐empirical arguments in favor of rotating multivariate solutions toward a hierarchical model.

#### Reducing stigma

First, psychiatric diagnoses are potentially stigmatizing. In the personality pathology domain, Wright and Hopwood argued that labeling patients' personality as disordered might be morally problematic (Wright & Hopwood, 2022). Instead, they suggested that it would be more ethical to use hierarchical models to describe patients' overall functioning separately from their personality traits.

On a somewhat similar note, there is a trend to re‐conceptualize neurodevelopmental disorders, such as autism and ADHD, as neurodiverse conditions, which involves recognizing not only their challenges but also their relative strengths (Baron‐Cohen, 2017). This is a difficult argument to make from an epidemiological perspective because individuals diagnosed with these conditions tend to have higher probabilities of many clinically relevant outcomes. However, specific factors unrelated to general psychopathology tend to display favorable and unfavorable correlates at both dimension poles. For example, as evident by the PC rotation in Figure 3, once general psychopathology was isolated, externalizing problems protected against later depression (as indicated by antidepressant prescription). Likewise, in analyses of self‐rated mood, personality, and personality problems, we observed that specific factors unrelated to general psychopathology displayed positive and negative attributes at both dimensional poles (Pettersson et al., 2012; Pettersson & Turkheimer, 2013, 2014). For instance, individuals who self‐reported as anxious also claimed to abstain from anti‐social behaviors; extraverted individuals endorsed traits related to anger and narcissism; and driven individuals endorsed traits related workaholism and perfectionism (Pettersson et al., 2014). Thus, although all rotations are mathematically arbitrary, a rotation toward a hierarchical model might reduce potential stigma by avoiding labeling personalities as disordered, and by highlighting that (general factor‐residualized) extreme trait scores seem associated with both favorable and unfavorable outcomes.

#### Unusual psychometric property of self‐reported general psychopathology

A second non‐empirical argument for rotating solutions toward a hierarchical model is that “items loading positively on p may have different and at times opposite meanings” (pp. 6.13; Smith et al., 2020). This was originally discovered by Edwards, who argued in the 1950s and 1960s that the first principal component of the MMPI did not capture anxiety (the predominant view at the time), but rather social desirability (Edwards & Heathers, 1962). Edwards had participants self‐report on a set of quadruples of items matched for both valence (i.e., perceived goodness vs. badness) and trait level (Edwards, 1969). For example, one quadruple set included the items *stingy* (bad, low trait), *thrifty* (good, low trait), *generous* (good, high trait), and *extravagant* (bad, high trait). He then extracted the first principal component from this item set and observed that it captured valence but ignored trait level. For example, individuals scoring low on the first principal component endorsed items such as *stingy* and *extravagant*, *inhibited* and *impulsive*, *tense* and *lethargic*, *self‐disparaging* and *conceited*, *distrustful* and *gullible*, *uncooperative* and *conforming*, and *domineering* and *submissive*, etc (Edwards, 1969). We replicated this observation in the domains of mood, personality, and personality problems (Pettersson et al., 2012, 2014; Pettersson & Turkheimer, 2013). While single pairs of opposite items might describe a consistent behavioral style (e.g., some individuals act in a domineering fashion toward their subordinates but submissively toward their superiors), it seems difficult to conjure up a behavioral style that fits all opposite markers simultaneously. Because a self‐rated general factor clusters symptoms of similar valence regardless of content but also predicts clinically relevant outcomes such as suicide attempts, criminal convictions, and overdoses, there might be merit in measuring this dimension in isolation from other variance.

## CONCLUSION

As part of the intake assessment, treatment seeking individuals at the psychotherapy clinic where I completed my graduate training filled out long questionnaires about their mental health. These individuals often appeared to score high on most scales, rendering it difficult to identify a core set of presenting issues. This might have occurred because many test developers adhere to Thurstone's simple structure principles. While those principles tend to generate factors that are intuitive to interpret, they can also obscure differences between individuals with mental health problems, limiting discriminant validity.

An alternate approach might be to develop tests based on hierarchical models. Rather than relying on unit‐weighted sum scores, such tests could use weighted scores designed retain the same correlations among the observed dimensions as the corresponding latent factors (ten Berge et al., 1999). Although such a weighting system sacrifices maximal reliability, the advantage is that the observed scores retain the key feature of hierarchical models, namely, the opportunity to measure general psychopathology independently of specific factors. Had I instead administered such a test at intake, I might have obtained a reliable measure of presenting individuals' overall distress and impairment. Furthermore, I might have more easily identified the primary ways in which they differed from their peers, which in turn could have better guided treatment choices.

## AUTHOR CONTRIBUTION

**Erik Pettersson**: Conceptualization; methodology; analysis; writing, and editing.

## CONFLICT OF INTEREST STATEMENT

The author has declared that he has no competing or potential conflicts of interest.

## ETHICAL CONSIDERATIONS

No ethical approval was required for this research review.

## Supporting information

Supporting Information S1

## Data Availability

The data are not publicly available due to privacy restrictions. Twin data can be applied for at the Swedish Twin Register. Data on the Swedish population can be applied for at the National Board of Health and Welfare.
