# Supplementary material for: Opportunities of measuring hierarchical models of psychopathology
Source: JCPP Adv. 2023 Jul 22;3(4):e12187. doi: 10.1002/jcv2.12187 (PMC10694532; doi:10.1002/jcv2.12187)
Supplement: Supplementary file 1 — Supporting Information S1 [file JCV2-3-e12187-s001.docx]

**Online appendix**

1. Conditioning on a precursor of an exposure
2. Conditioning on a collider (selection bias)
3. Examining degree simple structure among psychiatric conditions in child, adolescent, and adult samples

**Appendix S1. Conditioning on a precursor of an exposure**

As outlined by Pearl (<https://doi.org/10.1515/jci-2013-0003>), assuming the casual structure in the figure below, conditioning on a precursor of an exposure (z) does not change the association between an exposure (x) and an outcome (y). That is, if the below figure captures the true causal mechanism, regressing y on both x and z in a multiple regression would not change the association between x and y.


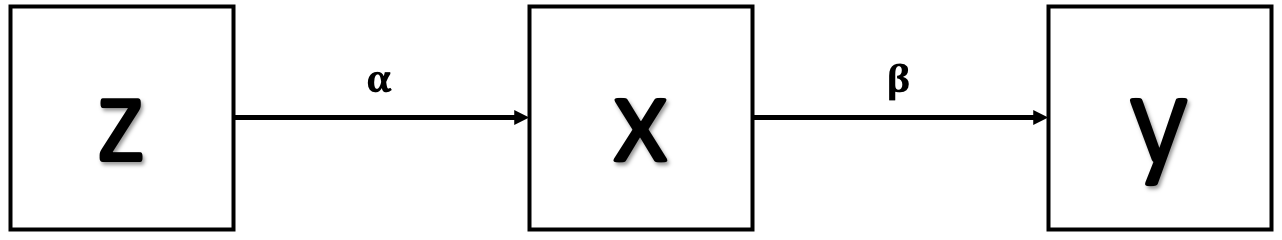


Below is the corresponding algebra for standardized variables.

$$\beta_{xy.z}=\frac{\beta_{yx}-\beta_{yz}*\beta_{zx}}{1-\beta_{zx}^{2}}$$

$$\beta_{xy.z}=\frac{\beta-(\alpha\beta*\alpha)}{1-\alpha^{2}}$$

$$\beta_{xy.z}=\frac{\beta-(\beta\alpha^{2})}{1-\alpha^{2}}$$

$$\beta_{xy.z}=\frac{\beta(1-\alpha^{2})}{1-\alpha^{2}}$$

$$\beta_{xy.z}=\beta$$

**Appendix S2.** **Conditioning on a collider (selection bias)**

As outlined by Pearl (<https://doi.org/10.1515/jci-2013-0003>), if the below figure captures the causal structure, then conditioning on collider z in the below figure changes the covariances among variables x and y.


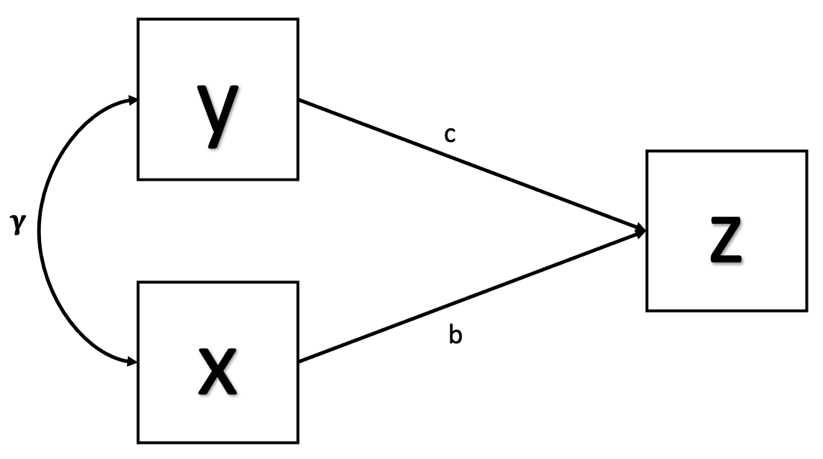


The below covariance algebra identifies the partial correlation between x and y, following stratifying / controlling for z.^[[1]](#footnote-1)^

$$r_{xy.z}=\frac{r_{yx}-r_{yz}*r_{zx}}{\sqrt{\left( 1-r_{xz}^{2} \right)\left( 1-r_{yz}^{2} \right)}}$$

$$r_{xy.z}=\frac{\gamma-\left( \left[ \gamma b+c \right]\left[ b+\gamma c \right] \right)}{\sqrt{{(1-\left[ b+\gamma c \right]}^{2}){(1-\left[ c+\gamma b \right]}^{2})}}$$

$$r_{xy.z}=\frac{\gamma-\left( \left[ \gamma bb+\gamma\gamma bc+cb+\gamma cc \right] \right)}{\sqrt{{(1-\left[ b+\gamma c \right]}^{2}){(1-\left[ c+\gamma b \right]}^{2})}}$$

$$r_{xy.z}=\frac{\gamma-\gamma bb-\gamma\gamma bc-cb-\gamma cc}{\sqrt{{(1-\left[ b+\gamma c \right]}^{2}){(1-\left[ c+\gamma b \right]}^{2})}}$$

$$r_{xy.z}=\frac{\gamma\left( 1-b^{2}-c^{2}-\gamma bc \right)-cb}{\sqrt{{(1-\left[ b+\gamma c \right]}^{2}){(1-\left[ c+\gamma b \right]}^{2})}}$$

**Appendix S3. Examining degree simple structure among psychiatric conditions in child, adolescent, and adult samples**

I used the “COMPLEXITY” function in the *EFA.dimensions* R-package (<https://cran.r-project.org/package=EFA.dimensions>) to compare the degree complexity among psychiatric conditions in child, adolescent, and adult samples. These samples are described elsewhere (<https://onlinelibrary.wiley.com/doi/full/10.1002/wps.20763>). Briefly, the adult sample included all Swedish individuals born between 1969 and 1979 (*N* = 909,699) such that they were between 35 and 45 years old at end of follow-up, when we examined if they had ever been diagnosed with 14 psychiatric diagnoses following contact with the in- or outpatient mental health care system. The adolescent sample consisted of 2,069 16-year olds who filled out the Child-Behavior Check-List. The child sample consisted of 14,589 9-year olds who were rated by their parents on scales related to neurodevelopmental, behavioral, and internalizing conditions.

The COMPLEXITY R-function generates a simulated multivariate solution based on an original solution (i.e., same number of indicators, communality, and dimensionality) where the indicators are randomly distributed in the multivariate space, before iteratively pushing the solution toward simple structure. The iteration starts at perfect complexity (100% complexity) and ends at perfect simple structure (0% complexity). At each iteration, the complexity of the solution is computed via Hoffman’s complexity index. Because the simulated solution contains randomly distributed variables, the process is aggregated over 100 runs at each complexity increment. Finally, Hoffman’s complexity index in the original solution is compared against the iterated simulated solution, providing an estimate for its degree complexity. The results are displayed in eTables 1-3 below.

| **Table S1.** Exploratory factor analysis of fourteen psychiatric diagnoses with Varimax rotation in 35-45 year olds (N = 909,699). | | | | | | | | | |
| --- | --- | --- | --- | --- | --- | --- | --- | --- | --- |
|  | 2-factor solution | | 3-factor solution | | | 4-factor solution | | | |
| Diagnosis | Int | Ext | Int | Ext | Psy | Int | Ext | Neuro | Psy |
| Depression | **0.85** | 0.29 | **0.78** | **0.34** | 0.26 | **0.77** | **0.31** | 0.23 | 0.27 |
| Anxiety | **0.76** | **0.33** | **0.70** | **0.38** | 0.23 | **0.66** | **0.36** | **0.32** | 0.15 |
| Obsessive-compulsive disorder | **0.64** | 0.20 | **0.61** | 0.08 | 0.36 | **0.53** | 0.07 | **0.47** | 0.18 |
| Post-traumatic stress disorder | **0.70** | 0.24 | **0.67** | **0.35** | 0.08 | **0.66** | **0.33** | 0.12 | 0.11 |
| Eating disorder | **0.59** | 0.11 | **0.55** | 0.15 | 0.15 | **0.53** | 0.13 | 0.20 | 0.12 |
| Bipolar disorder | **0.37** | **0.63** | 0.29 | **0.65** | 0.17 | 0.29 | **0.65** | 0.08 | 0.21 |
| Alcohol use | **0.44** | **0.73** | 0.27 | **0.89** | 0.24 | 0.27 | **0.85** | 0.17 | 0.24 |
| Drug abuse | **0.56** | **0.52** | **0.45** | **0.46** | **0.46** | **0.37** | **0.48** | **0.53** | 0.18 |
| Oppositional defiant/conduct disorder | 0.29 | **0.45** | 0.19 | **0.40** | **0.32** | 0.15 | **0.40** | 0.29 | 0.22 |
| Attention-deficit/hyper-activity disorder | **0.47** | **0.45** | **0.39** | 0.18 | **0.62** | 0.22 | 0.16 | **0.79** | 0.29 |
| Autism spectrum disorders | **0.44** | **0.35** | **0.34** | 0.06 | **0.60** | 0.20 | 0.13 | **0.68** | 0.12 |
| Tics | **0.61** | **0.42** | **0.55** | 0.28 | **0.45** | **0.55** | 0.24 | 0.19 | **0.49** |
| Schizoaffective disorder | 0.21 | **0.85** | 0.18 | 0.19 | **0.91** | 0.24 | 0.14 | 0.09 | **0.89** |
| Schizophrenia | 0.16 | **0.73** | 0.09 | 0.28 | **0.75** | 0.04 | 0.23 | 0.29 | **0.79** |
| RMSEA (90% CI) | 0.013 (0.013, 0.013) | | 0.009 (0.009, 0.010) | | | 0.007 (0.007, 0.007) | | | |
| CFI | 0.986 | | 0.994 | | | 0.997 | | | |
| TLI | 0.980 | | 0.989 | | | 0.994 | | | |
| $\chi^{2}$(df, *p*) | 9760.822 (64, *p* < 0.001) | | 4260.527 (52, *p* < 0.001) | | | 1875.190 (41, *p* < 0.001) | | | |
| Degree complexity | 88% | | 85% | | | 81% | | | |
| *Note.* Loadings equal to or greater than \|0.30\| are bolded. Int = Internalizing conditions. Ext = Externalizing conditions. Psy = Psychotic conditions. Neuro = Neurodevelopmental conditions. | | | | | | | | | |

| **Table S2.** Exploratory factor analysis of eight Child Behavior Checklist scales with Varimax rotation in 16 year olds (*N* = 2,069). | | | | | |
| --- | --- | --- | --- | --- | --- |
|  | 2-factor solution | | 3-factor solution | | |
| Scale | Internalizing conditions | Externalizing conditions | Internalizing conditions | Externalizing conditions | Withdrawn conditions |
| Anxious/depressed | **0.70** | 0.08 | **0.59** | 0.05 | **0.50** |
| Somatic complaints | **0.73** | 0.14 | **0.46** | 0.10 | **0.57** |
| Thought problems | **0.58** | 0.24 | **0.71** | 0.23 | 0.19 |
| Withdrawn behavior | **0.70** | **0.33** | 0.17 | 0.25 | **0.89** |
| Social problems | **0.56** | **0.32** | **0.49** | **0.30** | **0.32** |
| Attention problems | **0.42** | **0.72** | 0.16 | **0.69** | **0.45** |
| Rule-breaking | 0.01 | **0.91** | 0.00 | **0.88** | 0.12 |
| Aggression | 0.22 | **0.86** | 0.22 | **0.88** | 0.13 |
| RMSEA (90% CI) | 0.044 (0.038, 0.050) | | 0.037 (0.031, 0.044) | | |
| CFI | 0.987 | | 0.992 | | |
| TLI | 0.963 | | 0.973 | | |
| $\chi^{2}$(df, *p*) | 208.159 (42, *p* <0.001) | | 139.431 (36, *p* < 0.001) | | |
| Degree complexity | 72% | | 82% | | |
| *Note.* Loadings equal to or greater than \|0.30\| are bolded. | | | | | |

| **Table S3.** Exploratory factor analysis of fourteen parent-rated scales with Varimax rotation in 9-year old children (*N* = 14,589). | | | | | | | | | |
| --- | --- | --- | --- | --- | --- | --- | --- | --- | --- |
|  | 2-factor solution | | 3-factor solution | | | 4-factor solution | | | |
| Scale | Ext | Neuro | Neuro | Anx | Ext | Neuro | OCD | Ext | Anx |
| Inattention | **0.31** | 0.24 | **0.38** | 0.02 | **0.30** | **0.38** | 0.08 | 0.29 | 0.02 |
| Learning problems | **0.38** | **0.47** | **0.53** | 0.22 | **0.35** | **0.49** | 0.25 | **0.34** | 0.17 |
| Memory problems | **0.86** | 0.27 | 0.10 | **0.33** | **0.86** | 0.12 | 0.11 | **0.83** | **0.35** |
| Executive function problems | **0.54** | **0.47** | 0.11 | **0.58** | **0.50** | 0.15 | 0.11 | **0.46** | **0.60** |
| Impulsivity | **0.62** | 0.11 | 0.29 | -0.04 | **0.63** | 0.24 | 0.15 | **0.64** | -0.07 |
| Oppositional problems | **0.61** | **0.41** | 0.20 | **0.43** | **0.57** | 0.22 | 0.13 | **0.54** | **0.44** |
| Conduct problems | **0.84** | 0.17 | 0.20 | 0.15 | **0.81** | 0.17 | 0.15 | **0.81** | 0.14 |
| Sensory problems | **0.52** | **0.43** | **0.49** | 0.21 | **0.49** | **0.52** | 0.13 | **0.47** | 0.19 |
| Language problems | **0.49** | **0.61** | **0.44** | **0.47** | **0.45** | **0.45** | 0.22 | **0.42** | **0.44** |
| Inflexibility | **0.38** | **0.68** | **0.53** | **0.47** | **0.34** | **0.53** | 0.27 | **0.31** | **0.42** |
| Motor problems | 0.20 | **0.39** | 0.26 | **0.30** | 0.17 | 0.27 | 0.13 | 0.15 | 0.28 |
| Social problems | 0.09 | **0.42** | 0.34 | 0.25 | 0.07 | 0.26 | **0.32** | 0.07 | 0.17 |
| Anxiety | **0.38** | **0.59** | 0.21 | **0.65** | **0.32** | 0.18 | **0.31** | 0.29 | **0.59** |
| Mood problems | **0.31** | **0.43** | 0.12 | **0.50** | 0.26 | 0.11 | 0.18 | 0.24 | **0.47** |
| Obsessive-compulsive problems | 0.24 | **0.51** | **0.40** | **0.34** | 0.21 | 0.20 | **0.68** | 0.21 | 0.16 |
| Tics | 0.29 | **0.47** | 0.26 | **0.42** | 0.26 | 0.08 | **0.54** | 0.25 | **0.30** |
| RMSEA (90% CI) | 0.081 (0.080, 0.081) | | 0.063 (0.061, 0.064) | | | 0.052 (0.050, 0.054) | | | |
| CFI | 0.914 | | 0.957 | | | 0.975 | | | |
| TLI | 0.884 | | 0.931 | | | 0.952 | | | |
| $\chi^{2}$(df, *p*) | 8657.143 (89, *p* < 0.001) | | 4357.410 (75, *p* < 0.001) | | | 2520.210 (62, *p* < 0.001) | | | |
| Degree complexity | 96% | | 97% | | | 91% | | | |
| *Note.* Loadings equal to or greater than \|0.30\| are bolded. Ext = Externalizing conditions. Neuro = Neurodevelopmental conditions. Anx = Anxiety conditions. OCD = Obsessive-compulsive conditions. | | | | | | | | | |

1. Note that equation #20 in Pearl (2013) erroneously appeared to omit the term *yybc*. [↑](#footnote-ref-1)
